# Trajectories of Adherence to Biologic Disease-Modifying Anti-Rheumatic Drugs in Tuscan Administrative Databases: The Pathfinder Study

**DOI:** 10.3390/jcm10245743

**Published:** 2021-12-08

**Authors:** Irma Convertino, Sabrina Giometto, Rosa Gini, Massimiliano Cazzato, Marco Fornili, Giulia Valdiserra, Emiliano Cappello, Sara Ferraro, Claudia Bartolini, Olga Paoletti, Silvia Tillati, Laura Baglietto, Giuseppe Turchetti, Leopoldo Trieste, Valentina Lorenzoni, Corrado Blandizzi, Marta Mosca, Marco Tuccori, Ersilia Lucenteforte

**Affiliations:** 1 Unit of Pharmacology and Pharmacovigilance, Department of Clinical and Experimental Medicine, University of Pisa, 56126 Pisa, Italy; convertino.irma@gmail.com (I.C.); giuliavaldiserra@gmail.com (G.V.); emilianocappello@gmail.com (E.C.); saraferraro.a@gmail.com (S.F.); c.blandizzi@gmail.com (C.B.); marco.tuccori@gmail.com (M.T.); 2 Unit of Medical Statistics, Department of Clinical and Experimental Medicine, University of Pisa, 56126 Pisa, Italy; sabrina.giometto@med.unipi.it (S.G.); marco.fornili@med.unipi.it (M.F.); silvia.tillati@med.unipi.it (S.T.); laura.baglietto@unipi.it (L.B.); 3 Unit of Pharmacoepidemiology, Tuscan Regional Healthcare Agency, 50100 Florence, Italy; rosa.gini@ars.toscana.it (R.G.); claudia.bartolini@ars.toscana.it (C.B.); olga.paoletti@ars.toscana.it (O.P.); 4 Unit of Rheumatology, University Hospital of Pisa, 56100 Pisa, Italy; maxxmed@live.com (M.C.); marta.mosca@med.unipi.it (M.M.); 5 Institute of Management, Scuola Superiore Sant’Anna, 56127 Pisa, Italy; giuseppe.turchetti@santannapisa.it (G.T.); l.trieste@sssup.it (L.T.); v.lorenzoni@sssup.it (V.L.); 6 Unit of Adverse Drug Reactions Monitoring, University Hospital of Pisa, 56100 Pisa, Italy

**Keywords:** adherence, biologic, DMARD, real world evidence, rheumatoid arthritis

## Abstract

Scanty information on clustering longitudinal real-world data is available in the medical literature about the adherence implementation phase in rheumatoid arthritis (RA). To identify and characterize trajectories by analyzing the implementation phase of adherence to biologic Disease-Modifying Anti-Rheumatic Drugs (DMARDs), we conducted a retrospective cohort drug-utilization study using Tuscan administrative databases. RA patients were identified by a validated algorithm, including the first biologic DMARD supply from 2010 to 2015, RA specialist visit in the year before or after the first supply date and RA diagnosis in the five years before or in the year after the first supply date. We observed users for three years or until death, neoplasia, or pregnancy. We evaluated adherence quarterly through the Medication Possession Ratio. Firstly, we identified adherence trajectories and described the baseline characteristics; then, we focused on the trajectory most populated to distinguish the related sub-trajectories. We identified 952 first ever-biologic DMARD users in RA (712 females, mean age 52.7 years old, standard deviation 18.8). The biologic DMARD mostly supplied was etanercept (387 users) followed by adalimumab (233). Among 935 users with at least 3 adherence values, we identified 49 fully-adherent users, 829 continuous users, and 57 early-discontinuing users. Significant differences were observed among the index drugs. After focusing on the continuous users, three sub-trajectories were identified: continuous-steady users (556), continuous-alternate users (207), and continuous-declining users (66). No relevant differences emerged at the baseline. The majority of first ever-biologic DMARD users showed a continuous adherence behavior in RA. The role of adherence potential predictors and the association with effectiveness and safety outcomes should be explored by further studies.

## 1. Introduction

Rheumatoid arthritis (RA) is an immune-mediated inflammatory disease (IMID) resulting in joint deformation and disability [1], with negative impact on quality of life [2,3]. Poor RA control can promote other diseases [4], including cardiovascular ones [5], which add to pre-existing comorbidities [6] and increase mortality [7]. RA requires a life-long treatment. The first-line pharmacological approach includes glucocorticoids, non-steroidal anti-inflammatory drugs (NSAIDs), opioids, as well as conventional disease-modifying anti-rheumatic drugs (DMARDs) [8,9,10]. When poor control of the disease occurs, biologic DMARDs are recommended as second line treatment [9,10]. Rheumatologists must continuously monitor the disease activity and adjust the treatment accordingly [10]. In this regard, monitoring drug utilization and adherence are crucial for verifying the appropriateness of use.

The use of administrative healthcare databases to assess drug utilization patterns and adherence has been increasing over the years [11,12,13]. Adherence can be investigated by evaluating the three interrelated distinguished phases in which it is defined by the recent guidelines [14,15]: initiation (from first prescription to first dispensation), implementation (from the first dispensation to first discontinuation), and discontinuation (end of supply) [14,15,16]. The traditional methods used to assess adherence in the implementation phases include the proportion of days covered (PDC) [17] and the medication possession ratio (MPR) [18], which condense a complex pattern of longitudinal adherence observations into a single value. On the contrary, longitudinal models (e.g., group-based trajectory models) can discriminate the different dynamic adherence experiences over time [19,20,21]. Many approaches to the assessment of adherence trajectory usually need to make assumptions on clusters, namely, adherence distribution or trajectory shapes [22]. To the best of our knowledge, longitudinal adherence in new users of biologic DMARDs was assessed only in psoriasis patients [21] and never in RA ones.

The pathfinder study [23] is a population-based study on Tuscan RA patients that investigates drug-utilization, adherence, effectiveness, and safety of biologic DMARDs from the perspective of the health authority. This paper, conducted in first ever users of biologic DMARDs, was aimed at identifying and describing trajectories of adherence to these drugs (implementation phase). Particularly, we have used a method proposed by Leffondre et al. [19], which does not need any assumptions on adherence thresholds or trajectory shapes.

## 2. Materials and Methods

### 2.1. Study Design and Participants

Italy, with about 60 million inhabitants, has a national, universal, single-payer, public health system, which covers the services dispensed to patients at the regional level. These are collected in the administrative healthcare databases [12]. We conducted a retrospective, population-based drug-utilization cohort study using administrative healthcare databases of Tuscany, a region in central Italy with 3.7 million inhabitants. This study was designed in accordance with current guidelines on conduction and reporting of adherence studies [14,15,16,24]. The study protocol was recorded in the European Network of Centres for Pharmacoepidemiology and Pharmacovigilance (ENCePP^®^) register obtaining the quality seal (EUPAS29263) [23] and approved by the Ethical Committee of Pisa University Hospital (Protocol number 18,724). The Agenzia Regionale di Sanità Toscana (ARST) manages the regional administrative databases collecting information from 2004. On 29 April 2021 we extracted data from 2004 to 31 December 2018 (study period). The databases include longitudinal pseudo-anonymized patient-level information on the healthcare service utilization. The databases, linked at an individual level, encompass primary and secondary care, and include demographic registry (with records of entries and exits from regional assistance coverage), supplies of drugs (identified by Anatomical Therapeutic Chemical, ATC code), exemptions from co-payments, hospital discharge and emergency department (ED) accesses, registry of specialist visits, childbirth assistance certificates (CAP), spontaneous abortion (SAB), and voluntary interruption of pregnancy (VIP). Information about dates of prescription is not available in these databases. Hospital discharge records encompass information on causes of hospitalization (International Classification of Diseases 9th edition, ICD-9) with one primary diagnosis (usually the main cause of hospitalization) and several secondary diagnoses (other patient-relevant co-morbidities). ED access registry includes information on the main cause of admission (ICD-9 codes). Exemption from co-payments codes is assigned to subjects with specific features (e.g., disease-related, age-related, income-related) for which the regional healthcare system provides full coverage of the cost of the services supplied.

Since the therapeutic indication of the drugs supplied was not recorded in the above-mentioned databases, we have defined RA patients on the basis of an algorithm previously validated [25]. Based on this algorithm, we selected the first ever users of biologic DMARDs (infliximab, adalimumab, certolizumab pegol, etanercept, golimumab, abatacept, tocilizumab, rituximab) through the related ATC codes (L04AB02, L04AB04, L04AB05, L04AB01, L04AB06, L04AA24, L04AC07), starting their treatment from 1 January 2010 to 31 December 2015 (inclusion period) and a visit in a rheumatology ward in the year before or after the date of the first ever biologic DMARD supply. We referred to the first ever supply date of one of these drugs as the index date (ID), and to the year before ID as the look-back period. The first ever user was defined by no biologic DMARD supplied in the period elapsing from the first record available in the database for that subject to the ID (washout period). This cohort, composed of patients using biologic DMARDs as first-line or second-line, was further restricted to those subjects with at least one record of RA diagnosis (the earliest available between the primary and secondary diagnoses) in the repositories of hospitalizations or ED accesses (ICD-9 codes 714 *) or RA-related exemption from co-payments, in the five years before or one year after the index date. We excluded subjects not resident in Tuscany, with a look-back period shorter than one year, and with rituximab as the index drug (since rituximab is approved for oncologic indications as well). We followed patients for three years censoring them for neoplasia (i.e., hospital or ED admissions with an ICD-9 codes of 140 *–239 * or with disease-related co-payment exemption code of 048 *) or pregnancy (we used the conception date recorded in CAP, SAB, and VIP databases, or the date of hospital or ED admission associated with pregnancy and related-complications, ICD-9 codes: 630 *–677 *) or death—whichever came first. A diagram showing the time window of the study is provided in Supplementary material (S) (Figure S1).

### 2.2. Measurement

We measured the adherence during the implementation phase. For each patient, we calculated one adherence measure for each of the 12 quarterly periods. Each adherence measure was calculated through the MPR, dividing the number of days covered by the supply for the number of days of observation (90 days). The number of days covered by the supply was calculated as the number of Defined Daily Doses (DDD) available at the WHO Collaborating Centre for Drug Statistics Methodology website at the time of the analyses [26]. The number of days covered by two or more supplies in the same trimester was added regardless of overlaps, assuming a period of over-supply as determined by a new supply made in advance of the end of coverage of the previous supply (see example showed in the Figure S2).

We considered the following categories of variables as baseline characteristics of patients: time invariant (age at ID and gender), and single event (calendar year of ID; history of selected diseases—lung disease, myocardial infarction, stroke, hypertension, other cardiovascular diseases, diabetes, fracture of hip or spine or leg, depression, gastrointestinal ulcer, other gastrointestinal disorders, Sjögren’s syndrome, rheumatoid nodules, myopathies, polyneuropathy, cancer, and additional IMIDs; selection of drugs of interest recorded in the year before the ID—glucocorticoids for systemic use, non-steroidal anti-inflammatory drugs, opioid analgesics, and conventional synthetic DMARDs).

### 2.3. Statistical Analysis

We first described the cohort identified by the algorithm according to baseline characteristics. Then, we selected patients with at least three consecutive non-missing adherence values in the follow-up and conducted adherence trajectory analyses. We identified a set of adherence trajectories for all patients in the first-phase analysis, then, we selected patients belonging to the most populated adherence trajectory and identified a second set of adherence sub-trajectories in the second-phase analysis.

After the identification of trajectories, spaghetti plots were used to visualize individual data of a random sample of 50 patients in each cluster, in order to explore intra- and inter-individual variability, which cannot be shown by summary measures, such as mean or median, over time. Then, we labelled trajectories according to adherence trends. Finally, we compared trajectories in terms of baseline characteristics using the chi-squared test or *t*-test, as appropriate.

Trajectories were identified using the method proposed by Leffondrè et al. [19,20], consisting of three-steps. In the first step, 24 statistical measures for the 12 adherence values for each patient were computed. These measures discriminate between crescent/decrescent patterns, linear/non-linear, monotone/non-monotone, and stable/unstable. A combination of all measures would completely describe the patterns of change, but it would give problems of redundancy, and thereby of interpretability. To calculate the measures, at least three consecutive non-missing values are required, thus, in this first step, we included only patients fulfilling this criterion. In the second step, a principal component analysis was conducted in order to select the subset of measures that explained the largest proportion of variability in the data. Starting from the correlation matrix of the original variables, eigenvalues (one for each original variable) were computed, and only values equal or greater than one were considered. The eigenvalue with the higher value corresponded to the variance of the first principal component; the second eigenvalue corresponded to the variance of the second principal component, and so on. For each eigenvalue, the correspondent eigenvector was computed, which is the vector of loadings that, multiplied by the original variables, gives the new components. This vector of loadings is the vector of rotation; the varimax rotation option was adopted in order to facilitate the interpretation of the results. The third step consisted in conducting a cluster analysis based on the k-means algorithm, which consists in an iterative system of assigning the observations to the nearest cluster by choosing the initial centroid of the k clusters, k random points, and modifying these centroids at each iteration recalculating the average value for each cluster. The number of clusters k is needed a priori, in order to perform the k-means method. The aim of this last step was to group patients with similar longitudinal trajectories. Since there is no consensus regarding the method for choosing the optimal number of clusters, we decided to calculate 30 statistical indexes and follow the majority rule, choosing the number of clusters, giving priority to those with the highest number of indexes, but considering drug utilization interpretability as well. These indexes were based on the within-group and between-group dispersion matrix and the sum of the within-cluster and between-cluster distances.

We performed a sensitivity analysis dropping the covered days of overlapping supplies in the same trimester (i.e., we adopted the PDC approach) and identifying a new set of trajectories in both the first- and second-phase analysis.

We used R, version 4.0.0, with the AdhereR package [27,28] to compute the adherence, the NbClust package to calculate the best number of clusters [29], the traj package [30] to identify the trajectories, and the ggplot2 package version 0.8.0 for spaghetti plots [31].

## 3. Results

**Figure 1:**
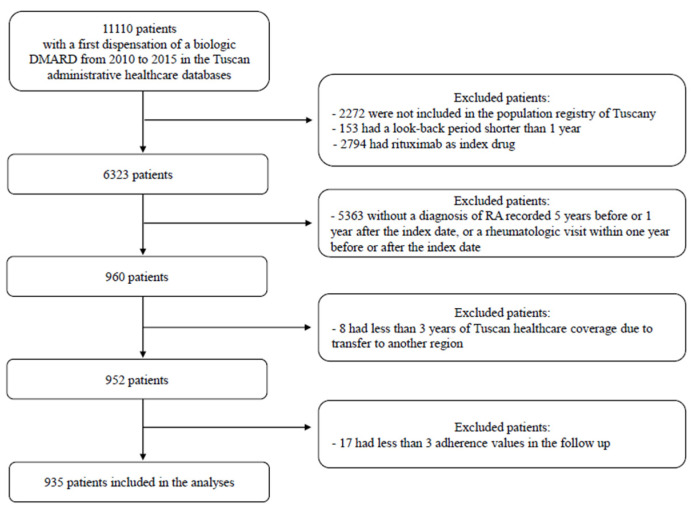
Study flow chart. DMARDs: Disease-modifying Anti-Rheumatic Drugs; RA: rheumatoid arthritis.

We identified 11,100 first users of biologic DMARDs between 2010 and 2015 in Tuscany. Overall, 6323 were included in the population registry, had a look-back period of at least 1 year, and had no rituximab as index drug. Among these, based on our validated algorithm [25], 960 were RA patients and 952 had at least 3 years of follow-up (Figure 1). Based on our data, the annual incidence of new users of biologic for RA in Tuscany is about 4.5 per 100,000 resident/years.

Table 1 summarizes the main characteristics of the study sample. The mean age was 52.7 years (standard deviation, SD 18.8) and 74.8% were females. The most frequent observed comorbidity was additional IMIDs (62 patients; 6.5%). The most frequently used concomitant therapies were conventional synthetic DMARDs (837; 87.9%), followed by glucocorticoids (757; 79.5%), and NSAIDs (628; 66.0%). Thirty-three patients (3.5%, data not shown) did not use conventional synthetic DMARDs or glucocorticoids in the year before ID, thus, they can be considered as the first-line users of biologic DMARDs. Patients started biologic DMARD treatment most frequently with etanercept (387; 40.7%) and adalimumab (233; 24.5%). Patients were followed for three years (887 patients) or until pregnancy (14), cancer (23), or death (28).

**Figure 2:**
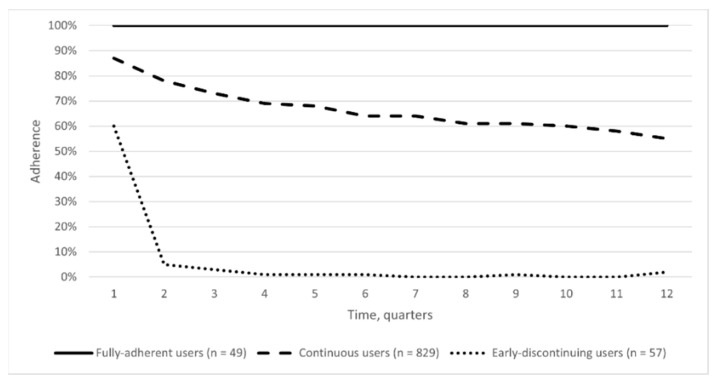
Adherence behavior to biologic DMARDs over three years of observation in the first-phase analysis.

The first-phase analysis, conducted on 935 patients with at least 3 consecutive adherence values, identified 3 trajectories of adherence to biologic DMARDs (Figure 2).

**Figure 3:**
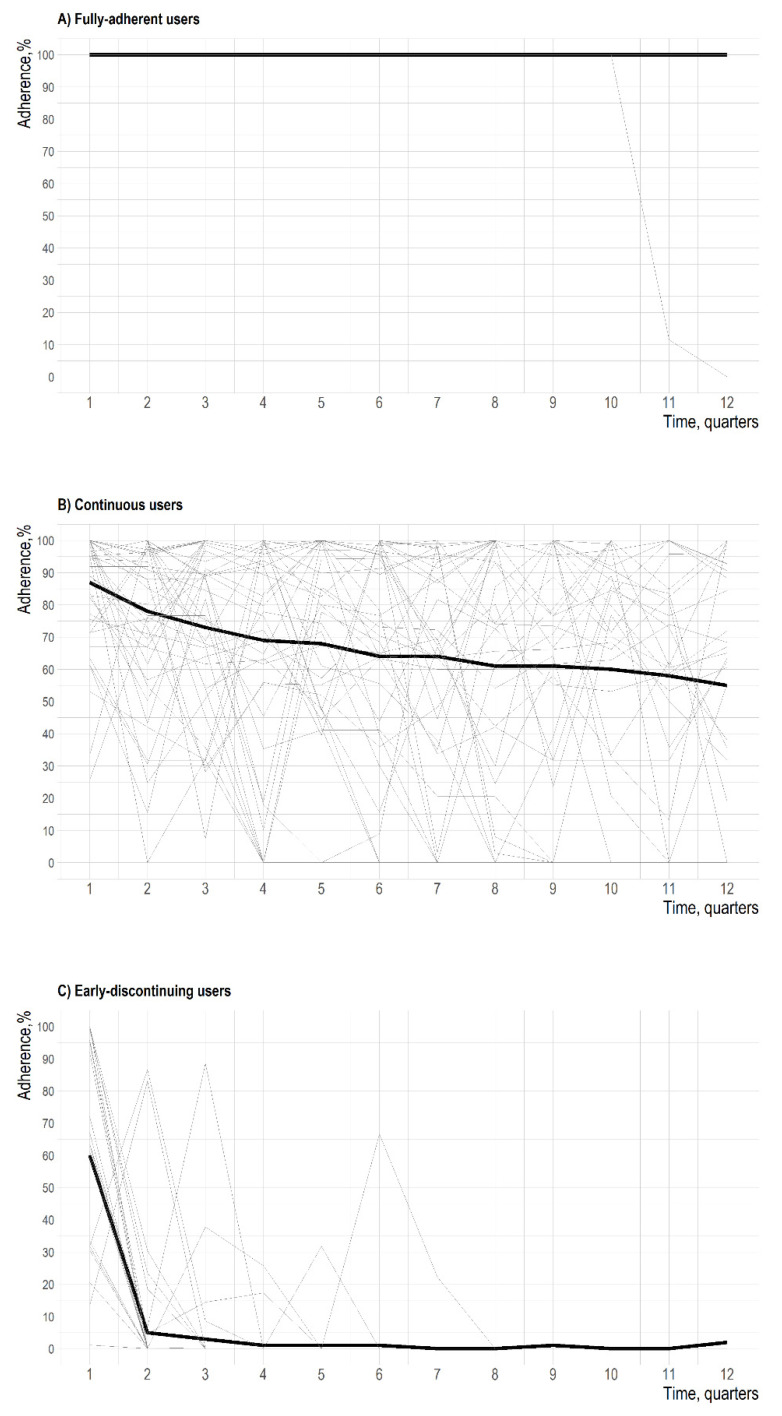
Spaghetti plots of random samples of 50 patients selected from each of the three adherence trajectories. Individual and mean trajectories are displayed in grey and black, respectively.

Figure 3 describes the spaghetti plot of each trajectory. Based on these findings, we decided to label users in each trajectory as fully-adherent users (49 patients), continuous users (829 patients), and early-discontinuing users (57 patients). In the fully-adherent users’ trajectory, we observed a mean adherence of 100% throughout the whole follow-up period with an homogeneous and constant adherence behavior over time of about all patients. In the continuous users’ one, the mean adherence decreased from 85% to 55% with high variability in the adherence behavior, characterized by a wide and heterogeneous range of adherence measures, fluctuating from peaks of full adherence to no- or low-adherence values. The early-discontinuing users’ trajectory was characterized by a quick loss of adherence up to the treatment discontinuation within 12 months from the index date. This trajectory includes users discontinuing treatment in few quarters with sporadic peaks of mean adherence below 80% (i.e., low adherence) in the first part of the follow-up period, and isolated supply of biologic DMARDs in the last part of observation period.

As far as index drug is concerned, abatacept was supplied more frequently as the index drug by continuous users, etanercept by continuous or early-discontinuing users, infliximab and certolizumab pegol by fully-adherent, and golimumab by early-discontinuing users. No significant difference was observed for the other baseline characteristics (Table 2), including no-history of any conventional synthetic DMARDs or glucocorticoids (data not shown).

When we considered the adherence data without taking into account the periods of overlaps (sensitivity analysis), the mean adherence in each trajectory inevitably dropped down. As a consequence, we obtained three new trajectories that were labelled as continuous users, occasional users, and early-discontinuing users. The three new trajectories have a trend that is not very different from that of the main analysis. The original continuous users’ trajectory remained the most populated. The occasional user’s trajectory encompasses those subjects with a rapid decrease of adherence in the first year and a subsequent sporadic use of biologic drugs (mean adherence <10%). The early-discontinuing users are those subjects discontinuing the treatment within three quarters (Figure S3). Among the index drugs, only golimumab was confirmed as the most supplied in the early-discontinuing trajectory in comparison with the main analysis, while the other baseline characteristics cardiovascular diseases were significantly distributed among the occasional users (Table S1).

**Figure 4:**
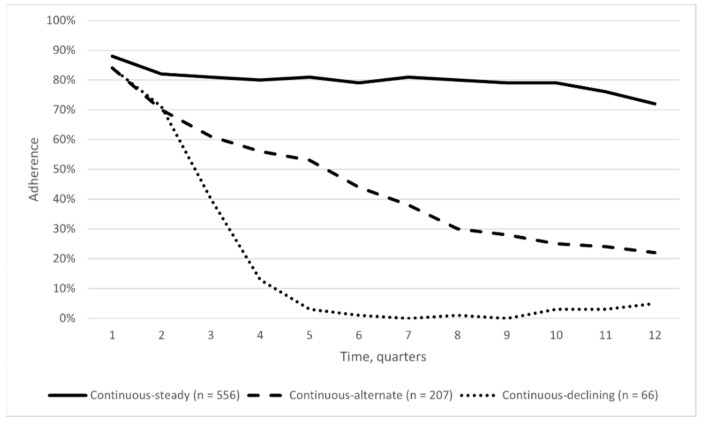
Sub-trajectories of adherence to biologic DMARDs in the second-phase analysis.

**Figure 5:**
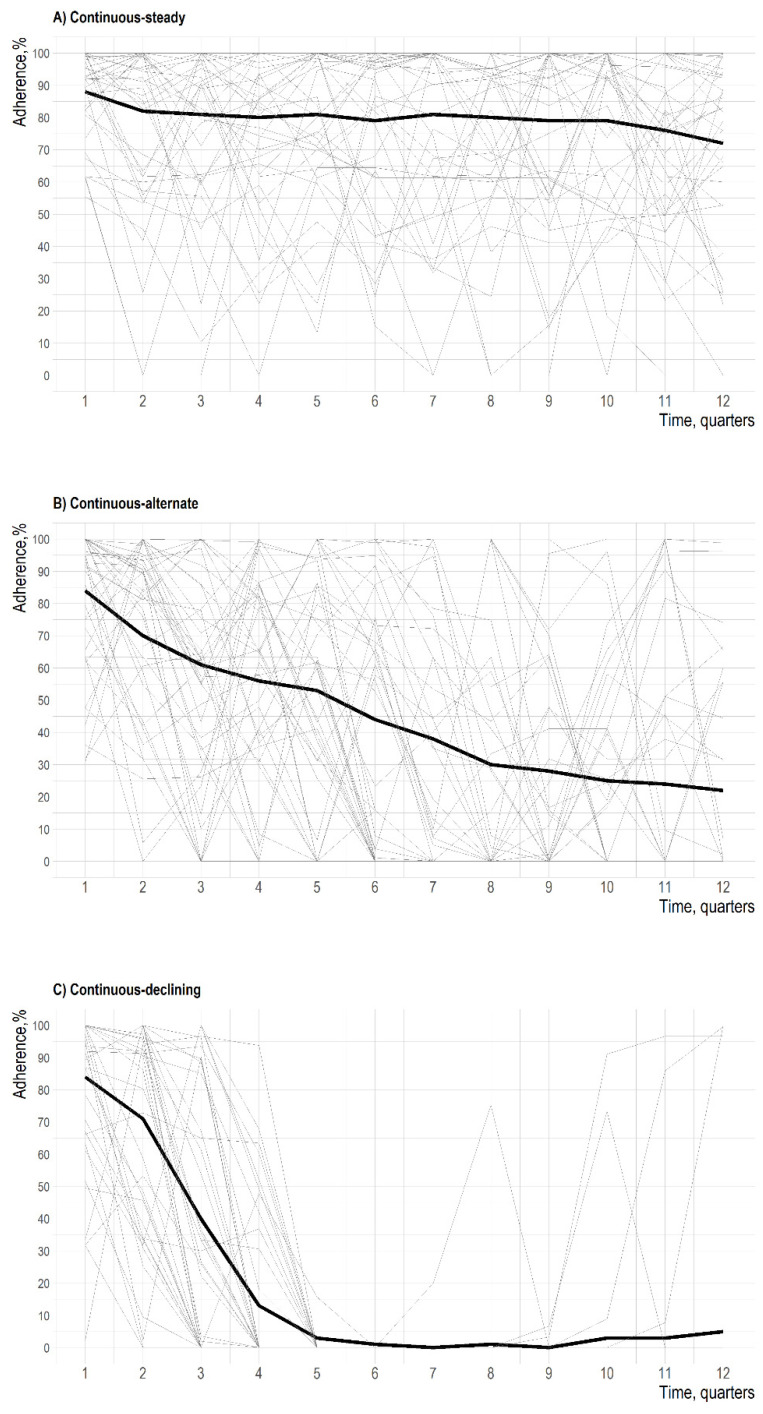
Spaghetti plot of random samples of 50 patients selected from each of the 3 adherence sub-trajectories identified in the second-phase analysis. Individual and mean sub-trajectories are displayed in grey and black, respectively.

In the second-phase analysis, we have separately analyzed the continuous users’ trajectory (829 patients) and identified a set of three adherence sub-trajectories: continuous-steady users (556 patients), continuous-alternate users (207 patients), and continuous-declining users (66 patients) (Figure 4 and Figure 5). During the follow-up, the continuous-steady users’ sub-trajectory showed a mean adherence over 70%. Users in this sub-trajectory showed only sporadic low- or no-adherence occurrences. The continuous-alternate users’ sub-trajectory displayed a progressive decrease of the mean adherence until about the 20% at the end of the observation. Users in this sub-trajectory alternate periods of high adherence with periods of low- or no-adherence to treatment. The continuous-declining users’ sub-trajectory is characterized by a progressive reduction in the mean adherence to biologic DMARDs, which reaches a minimum close to zero between 18 and 21 months and then remained below 10% up to the end of the follow-up. Users in this sub-trajectory are characterized by a fast decline in adherence in the first part of the follow up with single episodes of drug supplies in the second part. No relevant differences in baseline characteristics were observed among sub-trajectories (Table S2). The sensitivity analysis confirmed these results (Figure S4 and Table S3).

## 4. Discussion

Our study suggests that the model with three clusters optimally describes the adherence behavior over 3 years of observation of first-ever biologic DMARD RA users. The majority of patients (89%) belongs to continuous users’ trajectory. The remaining patients are fully-adherent users over time or early-discontinuers.

This study has several innovative elements that can be considered point of strengths. First, to the best of our knowledge, the implementation phase of adherence to biologic drugs in RA patients was never studied by means of trajectory models. Few studies tried to apply a trajectory model in RA patients, but they focused rather on disease activity scores or used different approaches to identify trajectories [32,33,34]. Second, since the method by Leffrondre et al. [19] does not require assumptions on clusters, it allowed the identification of several new categories of adherent patients to biologic DMARDs, who were previously poorly or not investigated [21,22,35]. Third, this is the first study performed in an Italian HAD in which RA patients using biologic drugs have been identified using a validated algorithm [25]. Fourth, we used data consolidated in previous population-based studies, since the Tuscan HAD had already been used as a data source for real world evidence [12,13].

Some limitations should be considered. First, given the nature of the data source, designed for administrative purposes, misclassification due to uncaptured exposures (private purchase or extra-regional supplying) could have occurred [36]. Therefore, prevalent users could have been rarely classified as first-ever users, explaining why, in few cases, non-anti TNF drugs, not recommended as first-line biologic therapy, were identified as index drugs [9]. Second, the data about drug exposure were taken from the drug reimbursement registry, and therefore patients may have received their drug supply without actually taking it. This could lead to an adherence overestimation. Third, the calculation of drug coverage was based on DDD and not on the prescribed daily dose. We cannot exclude that patients were adherent to their prescriber’s recommendations [14], which may have been tailored according to disease activity [9,10]. Fourth, the adherence computation might be influenced by the coverage definition, which included overlapping periods. The sensitivity analysis, conducted by dropping the overlapping periods, confirmed the trajectory trends. However, statistically significant differences among trajectories of baseline patients’ characteristics observed in the main analysis, were lost, thus confirming that some result should be interpreted with caution.

The anti-TNF drugs have been identified as index drugs more frequently than other biologic DMARDs, particularly etanercept (40.7%) and adalimumab (24.5%). These findings are in line with guidelines, supporting the use of these drugs as first-line biologics in RA, and with evidence from other coeval studies [37,38].

Available RA studies, which estimated adherence without considering the longitudinal adherence behavior over time (i.e., PDC), showed a great variability of results, with adherence ranging from 30% to 80% [39,40]. In our study, the majority of RA patients have a continuous adherence behavior to biologic DMARDs over time, with a certain variability in the supply of drugs, which could reflect both disease activity and therapeutic response. Indeed, since the aim of biologic DMARD treatment in RA is the achievement of a situation of in-target disease (i.e., DAS28 < 3.2) [9,10], the tapering or discontinuation of these drugs should represent clinical decisions in response to disease control needs [10,41] or safety problems [42,43].

Our analysis identified, for the first time, three adherence scenarios (trajectories), with three sub-trajectories for the most populated one. This allowed the identification of five major groups of drug users: fully-adherent, continuous-steady, continuous-alternate, continuous-declining and early-discontinuing. Fully-adherent and continuous-steady, representing 60% of the study population, are likely patients that require a continuous treatment to remain “in target”. Continuous-alternate and continuous-declining are subjects who alternate period of treatment with period of non-treatment. This situation likely reflects disease control needs or safety issues, which can be managed with frequent tapering or temporary suspension of treatments. The early-discontinuing group could include subjects that achieve long remission periods or with relevant tolerability problems. Further studies are needed to investigate in detail the relationship between drug-utilization behavior and effectiveness and safety clinical outcomes.

In the first-phase analysis, we observed some significant differences in the distribution of index drugs among trajectories. Infliximab was used more frequently by fully-adherent patients (26.5%) compared to continuous (2.3%) or early-discontinuing ones (8.8%). This is consistent with findings obtained in other real-world studies [37,39], and it could be explained by the route of administration of this drug (hospital intravenous administration), which allowed a closer monitoring of the fulfillment of the scheduled treatment [9,10] compared with home administered biologics. Certolizumab was also used more frequently by the fully-adherent group (24.5%) when compared with continuous (7.5%) or early-discontinuing one (3.5%). The interpretation and clinical implications of this is not clear and deserves further investigation. Abatacept was used more frequently by continuous users (10%) compared with fully-adherent (0%) or early-discontinuing users (3.5%). Abatacept was not recommended as first-line biologic in RA at the time of observation [44]. However, an Italian real-world study [45] demonstrated that abatacept is preferred as first-line biologic rather than anti-TNF in subject with specific co-morbidities, such as hypertension (39.13%) or pulmonary disorders (16.52%). The distribution of these co-morbidities in the trajectories identified in our study is not significantly different. Therefore, we have no evidence of such selective prescription. Finally, golimumab was the index drug mostly used by early-discontinuing users, whose adherence rapidly dropped down within the first two quarters. The mean adherence in the first quarter is 60% decreasing to 10% in the second quarter, and discontinuation occurred at one year. The GOAREL study, a prospective observational multicenter investigation, conducted in Italy on RA patients starting golimumab for the first time (with history of use of biologic drugs or not) suggested that no disease improvement in three months, according to EULAR recommendation, is a strong predictor of golimumab discontinuation (HR = 3.0, 95% CI: 1.26–7.30) [46]. Data from the Spanish biological drugs registry, BIOBADASER, showed that discontinuation after one year occurred in 29% of RA patients using golimumab as first-line therapy. Notably, one-year discontinuation rates in these patients increased to 54% and to 60% when golimumab is used as second- or third-line biologic, respectively [47]. In our study, the high frequency of golimumab observed in the early-discontinuing group is in line with results of the above-mentioned studies, and it could reflect a situation of lack of disease improvement in the first month of treatment. Notably, the sensitivity analysis confirmed this finding.

Considering the robustness of the methodology used, we are quite confident that our RA study cohort is representative of the Tuscan RA population. Despite that our findings could not be automatically extended to the entire Italian setting, due to differences in health administration among Italian Regions, even though we have analyzed the implementation phase of adherence through a different approach, and other methodological differences must be considered, many of our observations are in line with findings of the above-mentioned studies performed on RA patients in other Italian regions [45,46]. Moreover, the incident rate of RA patients starting biologic DMARDs analyzed in this study is in line with that reported in the National Report on Medicines use in Italy, (5–6 per 100,000 residents/year) [48,49].

## 5. Conclusions

In conclusion, this study, for the first time, characterized the first-ever-biologic DMARD RA users and clustered patients according to the real-world adherence behavior over time through trajectory approach. The majority of Tuscan RA patients belonged to the continuous users. Golimumab was the index biologic DMARD mostly observed in the early-discontinuing users. Further studies are warranted to investigate the trajectory predictors and the relationship between adherence behavior and effectiveness (disease activity indexes) and safety (adverse events) outcomes. Their results could provide new insight for the interpretation of the implementation phase of adherence to RA treatment and could be used by clinicians and health authority to improve the management of the disease.

## Figures and Tables

**Table 1:** Distribution of baseline characteristics of 952 new users of biologic DMARDs in 2010–2015 in Tuscany.

Baseline Characteristics	*n* (%)
Overall sample	952
Gender	
Female	712 (74.8)
Age, years	
mean (SD)	52.7 (18.8)
Categories	
0–20	90 (9.5)
21–40	117 (12.3)
41–50	143 (15.0)
51–60	227 (23.8)
61–70	218 (22.9)
71–80	136 (14.3)
81–100	21 (2.2)
Comorbidities	
Lung disease	17 (1.8)
Myocardial infarction	3 (0.3)
Stroke	6 (0.6)
Hypertension	27 (2.8)
Other CV diseases	35 (3.7)
Diabetes	29 (3.0)
Fractures	12 (1.3)
Depression	1 (0.1)
Gastrointestinal ulcer	0 (0.0)
Other gastrointestinal disorders	8 (0.8)
Sjögren’s syndrome	5 (0.5)
Rheumatoid nodules	0 (0.0)
Myopathies	1 (0.1)
Polyneuropathy	2 (0.2)
Cancer	13 (1.4)
Additional immune-mediated disorders	62 (6.5)
Concomitant therapies	
Glucocorticoid for systemic use	757 (79.5)
Non-Steroidal Anti-Inflammatory Drugs	628 (66.0)
Opioid analgesics	289 (30.4)
Conventional synthetic DMARDs	837 (87.9)
Index drug	
Abatacept	86 (9.0)
Etanercept	387 (40.7)
Infliximab	37 (3.9)
Adalimumab	233 (24.5)
Certolizumab pegol	79 (8.3)
Golimumab	66 (6.9)
Tocilizumab	64 (6.7)

CV: cardiovascular; DMARD: disease modifying antirheumatic drugs; *n*: number; SD: standard deviation.

**Table 2:** Distribution of baseline characteristics of 935 new users of biologic DMARDs in the first-phase analysis.

Baseline Characteristics	Trajectories			
	Fully-Adherent Users	Continuous Users	Early-Discontinuing Users	*p*-Value
Overall sample. n (%)	49	829	57	
Gender. n (%)				
Female	35 (71.4)	620 (74.8)	45 (78.9)	0.665
Age. years				
mean (SD)	51.8 (17.5)	52.3 (18.8)	57.5 (17.0)	0.114
Categories. n (%)				0.166
0–20	3 (6.1)	84 (10.1)	3 (5.3)	
21–40	9 (18.4)	100 (12.1)	5 (8.8)	
41–50	8 (16.3)	123 (14.8)	11 (19.3)	
51–60	14 (28.6)	200 (24.1)	10 (17.5)	
61–70	7 (14.3)	195 (23.5)	15 (26.3)	
71–80	7 (14.3)	114 (13.8)	9 (15.8)	
81–100	1 (2.0)	13 (1.6)	4 (7.0)	
Index date year. n (%)				0.660
2010	7 (14.3)	109 (13.1)	11 (19.3)	
2011	7 (14.3)	138 (16.6)	8 (14.0)	
2012	6 (12.2)	142 (17.1)	11 (19.3)	
2013	11 (22.4)	126 (15.2)	8 (14.0)	
2014	9 (18.4)	167 (20.1)	6 (10.5)	
2015	9 (18.4)	147 (17.7)	13 (22.8)	
Comorbidities. n (%)				
Lung disease	0 (0.0)	16 (1.9)	1 (1.8)	0.617
Myocardial infarction	0 (0.0)	2 (0.2)	0 (0.0)	0.880
Other CV diseases	2 (4.1)	28 (3.4)	3 (5.3)	0.740
Stroke	0 (0.0)	6 (0.7)	0 (0.0)	0.680
Hypertension	0 (0.0)	25 (3.0)	0 (0.0)	0.194
Diabetes	4 (8.2)	24 (2.9)	0 (0.0)	0.043
Fractures	0 (0.0)	10 (1.2)	0 (0.0)	0.524
Depression	0 (0.0)	1 (0.1)	0 (0.0)	0.938
Gastrointestinal ulcer	0 (0.0)	0 (0.0)	0 (0.0)	NA
Other gastrointestinal disorders	0 (0.0)	6 (0.7)	2 (3.5)	0.070
Sjögren’s syndrome	0 (0.0)	5 (0.6)	0 (0.0)	0.725
Rheumatoid nodules	0 (0.0)	0 (0.0)	0 (0.0)	NA
Myopathies	0 (0.0)	1 (0.1)	0 (0.0)	0.938
Polyneuropathy	0 (0.0)	2 (0.2)	0 (0.0)	0.880
Additional immune-mediated disorders	4 (8.2)	54 (6.5)	2 (3.5)	0.587
Cancer	1 (2.0)	11 (1.3)	1 (1.8)	0.891
Concomitant therapies. n (%)				
Glucocorticoid	42 (85.7)	657 (79.3)	43 (75.4)	0.417
Non-steroidal anti-inflammatory drugs	29 (59.2)	546 (65.9)	42 (73.7)	0.284
Opioid analgesic	18 (36.7)	246 (29.7)	15 (26.3)	0.482
Conventional synthetic DMARDs	43 (87.8)	728 (87.8)	50 (87.7)	1.000
Index drug. n (%)				
Abatacept	0 (0.0)	83 (10.0)	2 (3.5)	0.019
Etanercept	11 (22.4)	348 (42.0)	24 (42.1)	0.026
Infliximab	13 (26.5)	19 (2.3)	5 (8.8)	<0.001
Adalimumab	8 (16.3)	206 (24.8)	12 (21.1)	0.340
Certolizumab pegol	12 (24.5)	62 (7.5)	2 (3.5)	<0.001
Golimumab	1 (2.0)	56 (6.8)	8 (14.0)	0.043
Tocilizumab	4 (8.2)	55 (6.6)	4 (7.0)	0.914

CV: cardiovascular; DMARD: disease modifying antirheumatic drugs; n: number; SD: standard deviation.

## Data Availability

The data that support the findings of this study are available on request from the corresponding author.

## Supplementary Materials

The following are available online at https://www.mdpi.com/article/10.3390/jcm10245743/s1, Figure S1: Study design diagram; Figure S2: Example of adherence assessment estimated by the continuous medication availability; Figure S3: Trajectories of adherence to biologic DMARDs in the first-phase analysis (sensitivity analysis); Table S1: Distribution of baseline characteristics of 935 new users of biologic DMARDs in the first-phase analysis (sensitivity analysis); Table S2: Distribution of baseline characteristics of 829 new users of biologic DMARDs in the second-phase analysis; Figure S4: Sub-trajectories of adherence to biologic DMARDs in the second-phase analysis (sensitivity analysis); Table S3: Distribution of baseline characteristics of 829 new users of biologic DMARDs in the second-phase analysis (sensitivity analysis).
