# Supplementary material for: Trajectories of Adherence to Biologic Disease-Modifying Anti-Rheumatic Drugs in Tuscan Administrative Databases: The Pathfinder Study
Source: J Clin Med. 2021 Dec 8;10(24):5743. doi: 10.3390/jcm10245743 (PMC8708974; doi:10.3390/jcm10245743)
Supplement: Supplementary file 1 [file jcm-10-05743-s001.zip › Supplementary.pdf]

Supplementary Material

Trajectories of adherence to biologic disease-modifying anti-rheumatic drugs in Tuscan administrative databases: the Pathfinder study

Irma Convertino[1§], Sabrina Giometto[2§], Rosa Gini[3], Massimiliano Cazzato[4], Marco Fornili[2], Giulia Valdiserra[1], Emiliano Cappello[1], Sara Ferraro[1], Claudia Bartolini[3], Olga Paoletti[3], Silvia Tillati[2], Laura Baglietto[2], Giuseppe Turchetti[5], Leopoldo Trieste[5], Valentina Lorenzoni[5], Corrado Blandizzi[1,6], Marta Mosca[4], Marco Tuccori[1,6], Ersilia Lucenteforte[2*]

[1] Unit of Pharmacology and Pharmacovigilance, Department of Clinical and Experimental Medicine, University of Pisa, Pisa, Italy
[2] Unit of Medical Statistics, Department of Clinical and Experimental Medicine, University of Pisa, Pisa, Italy
[3] Tuscan Regional Healthcare Agency, Florence, Italy
[4] Unit of Rheumatology, University Hospital of Pisa, Pisa, Italy
[5] Institute of Management, Scuola Superiore Sant'Anna, Pisa, Italy
[6] Unit of Adverse Drug Reactions Monitoring, University Hospital of Pisa, Pisa, Italy

[§] co-first authors

[*] **Corresponding author**
Ersilia Lucenteforte
Associate Professor of Medical Statistics
Unit of Medical Statistics, Department of Clinical and Experimental Medicine, University of Pisa
Via Roma 67, 56126 Pisa
E-mail: ersilia.lucenteforte@unipi.it

Figure S1. Study design diagram 1

Figure S2. Example of adherence assessment estimated by the continuous medication availability. 2

Figure S3. Trajectories of adherence to biologic DMARDs in the first-phase analysis (sensitivity analysis). 3

Table S1. Distribution of baseline characteristics of 935 new users of biologic DMARDs in the first-phase analysis (sensitivity analysis). 4

Table S2. Distribution of baseline characteristics of 829 new users of biologic DMARDs in the second-phase analysis. 5

Figure S4. Sub-trajectories of adherence to biologic DMARDs in the second-phase analysis (sensitivity analysis). 6

Table S3. Distribution of baseline characteristics of 829 new users of biologic DMARDs in the second-phase analysis (sensitivity analysis). 7

Figure S1. Study design diagram

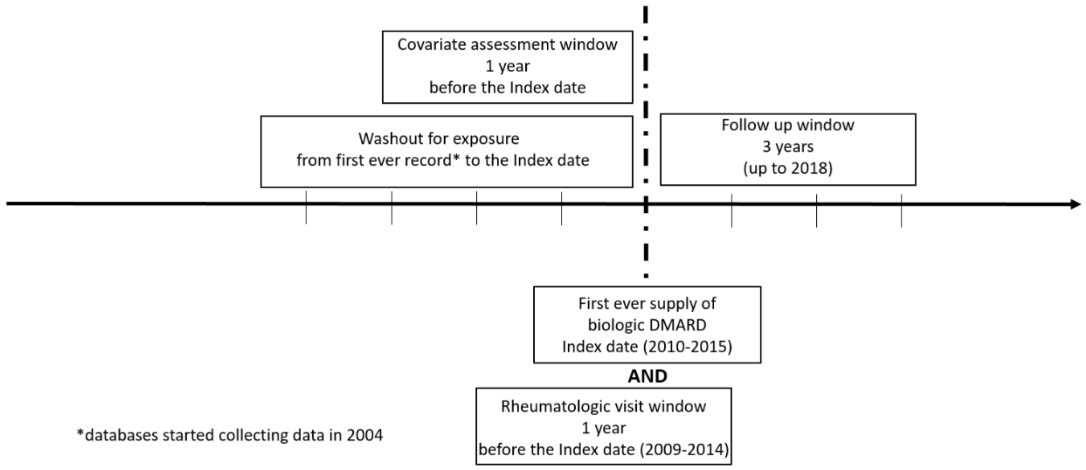

Figure S2. Example of adherence assessment estimated by the continuous medication availability.

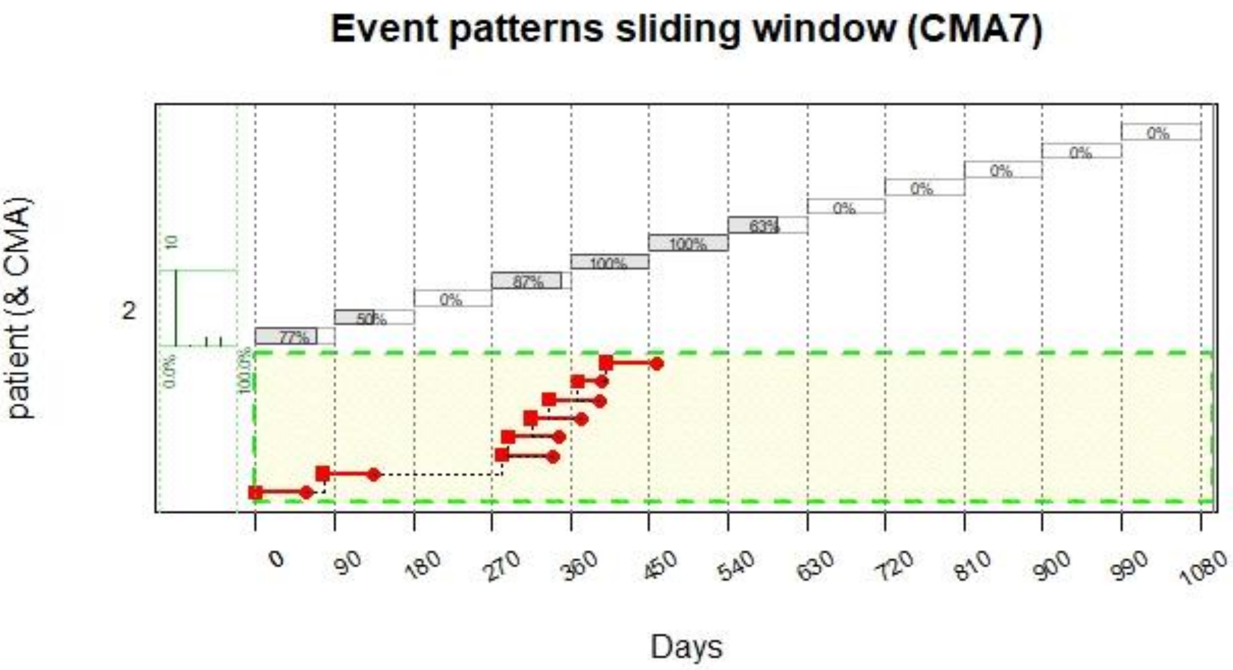

Figure S3. Trajectories of adherence to biologic DMARDs in the first-phase analysis (sensitivity analysis).

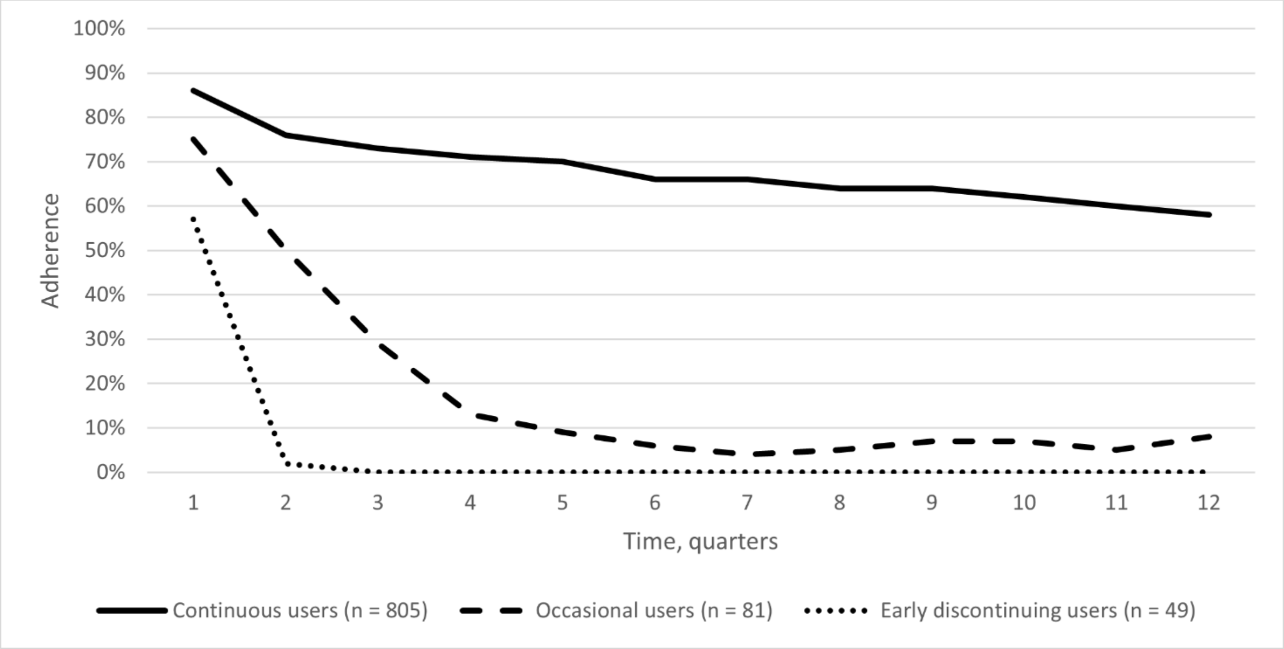

Table S1. Distribution of baseline characteristics of 935 new users of biologic DMARDs in the first-phase analysis (sensitivity analysis).

**Baseline Characteristics**	*Trajectories*			**p-value**
	**Continuous users**	**Progressively occasional use rs**	**Early-discontinuing users**	
Overall sample. n (%)	805	81	49	
Gender. n (%)				
Female	599 (74.4)	62 (76.5)	39 (79.6)	0.673
Age. years				
mean (SD)	51.9 (18.7)	55.5 (19.2)	58.2 (15.6)	0.022
Index date year. n (%)				0.373
2010	108 (13.4)	9 (11.1)	10 (20.4)	
2011	137 (17.0)	10 (12.3)	6 (12.2)	
2012	132 (16.4)	18 (22.2)	9 (18.4)	
2013	121 (15.0)	17 (21.0)	7 (14.3)	
2014	162 (20.1)	15 (18.5)	5 (10.2)	
2015	145 (18.0)	12 (14.8)	12 (24.5)	
Comorbidities. n (%)				
Lung disease	13 (1.6)	4 (4.9)	0 (0.0)	0.064
Myocardial infarction	2 (0.2)	0 (0.0)	0 (0.0)	0.851
Other CV diseases	21 (2.6)	9 (11.1)	3 (6.1)	<0.001
Stroke	6 (0.7)	0 (0.0)	0 (0.0)	0.614
Hypertension	21 (2.6)	4 (4.9)	0 (0.0)	0.228
Diabetes	26 (3.2)	2 (2.5)	0 (0.0)	0.418
Fractures	10 (1.2)	0 (0.0)	0 (0.0)	0.442
Depression	0 (0.0)	1 (1.2)	0 (0.0)	0.005
Gastrointestinal ulcer	0 (0.0)	0 (0.0)	0 (0.0)	NA
Other gastrointestinal disorders	6 (0.7)	1 (1.2)	1 (2.0)	0.587
Sjögren's syndrome	4 (0.5)	1 (1.2)	0 (0.0)	0.597
Rheumatoid nodules	0 (0.0)	0 (0.0)	0 (0.0)	NA
Myopathies	1 (0.1)	0 (0.0)	0 (0.0)	0.922
Polyneuropathy	2 (0.2)	0 (0.0)	0 (0.0)	0.851
Additional immune-mediated disorders	53 (6.6)	5 (6.2)	2 (4.1)	0.783
Cancer	11 (1.4)	1 (1.2)	1 (2.0)	0.919
Concomitant therapies. n (%)				
Glucocorticoid	644 (80.0)	63 (77.8)	35 (71.4)	0.332
Non-steroidal anti-inflammatory drugs	531 (66.0)	50 (61.7)	36 (73.5)	0.391
Opioid analgesic	238 (29.6)	30 (37.0)	11 (22.4)	0.191
Conventional synthetic DMARDs	706 (87.7)	73 (90.1)	42 (85.7)	0.735
Index drug. n (%)				
Abatacept	78 (9.7)	5 (6.2)	2 (4.1)	0.263
Etanercept	327 (40.6)	39 (48.1)	17 (34.7)	0.277
Infliximab	28 (3.5)	4 (4.9)	5 (10.2)	0.057
Adalimumab	199 (24.7)	16 (19.8)	11 (22.4)	0.584
Certolizumab pegol	69 (8.6)	5 (6.2)	2 (4.1)	0.427
Golimumab	50 (6.2)	7 (8.6)	8 (16.3)	0.021
Tocilizumab	54 (6.7)	5 (6.2)	4 (8.2)	0.905

CV: cardiovascular; DMARD: disease modifying antirheumatic drugs; n: number; SD: standard deviation

Table S2. Distribution of baseline characteristics of 829 new users of biologic DMARDs in the second-phase analysis.

**Baseline Characteristics**	*Sub-trajectories*			
	**Continuous-steady**	**Continuous-alternate**	**Continuous-declining**	***p*-value**
Overall sample. n (%)	556	207	66	
Gender. n (%)				
Female	414 (74.5)	153 (73.9)	53 (80.3)	0.554
Age. years				
mean (SD)	52.0 (18.5)	51.4 (20.1)	57.2 (17.3)	0.076
Index date year. n (%)				0.535
2010	77 (13.8)	23 (11.1)	9 (13.6)	
2011	97 (17.4)	34 (16.4)	7 (10.6)	
2012	87 (15.6)	42 (20.3)	13 (19.7)	
2013	81 (14.6)	30 (14.5)	15 (22.7)	
2014	112 (20.1)	45 (21.7)	10 (15.2)	
2015	102 (18.3)	33 (15.9)	12 (18.2)	
Comorbidities. n (%)				
Lung disease	9 (1.6)	4 (1.9)	3 (4.5)	0.263
Myocardial infarction	2 (0.4)	0 (0.0)	0 (0.0)	0.611
Other CV diseases	15 (2.7)	5 (2.4)	8 (2.1)	<0.001
Stroke	5 (0.9)	1 (0.5)	0 (0.0)	0.642
Hypertension	14 (2.5)	8 (3.9)	3 (4.5)	0.470
Diabetes	16 (2.9)	6 (2.9)	2 (3.0)	0.998
Fractures	7 (1.3)	2 (1.0)	1 (1.5)	0.920
Depression	0 (0.0)	0 (0.0)	1 (1.5)	0.003
Gastrointestinal ulcer	0 (0.0)	0 (0.0)	0 (0.0)	NA
Other gastrointestinal disorders	5 (0.9)	1 (0.5)	0 (0.0)	0.642
Sjögren's syndrome	2 (0.4)	1 (0.5)	2 (3.0)	0.029
Rheumatoid nodules	0 (0.0)	0 (0.0)	0 (0.0)	NA
Myopathies	1 (0.2)	0 (0.0)	0 (0.0)	0.782
Polyneuropathy	2 (0.4)	0 (0.0)	0 (0.0)	0.611
Additional immune-mediated disorders	36 (6.5)	15 (7.2)	3 (4.5)	0.739
Cancer	9 (1.6)	1 (0.5)	1 (1.5)	0.471
Concomitant therapies. n (%)				
Glucocorticoid	445 (80.0)	161 (77.8)	51 (77.3)	0.727
Non-steroidal anti-inflammatory drugs	368 (66.2)	137 (66.2)	41 (62.1)	0.800
Opioid analgesic	159 (28.6)	65 (31.4)	22 (33.3)	0.598
Conventional synthetic DMARDs	491 (88.3)	177 (85.5)	60 (90.9)	0.417
Index drug. n (%)				
Abatacept	57 (10.3)	22 (10.6)	4 (6.1)	0.531
Etanercept	227 (40.8)	96 (46.4)	25 (37.9)	0.301
Infliximab	11 (2.0)	5 (2.4)	3 (4.5)	0.416
Adalimumab	141 (25.4)	49 (23.7)	16 (24.2)	0.885
Certolizumab pegol	44 (7.9)	14 (6.8)	4 (6.1)	0.780
Golimumab	35 (6.3)	13 (6.3)	8 (2.1)	0.194
Tocilizumab	41 (7.4)	8 (3.9)	6 (9.1)	0.157

CV: cardiovascular; DMARD: disease modifying antirheumatic drugs; n: number; SD: standard deviation

Figure S4. Sub-trajectories of adherence to biologic DMARDs in the second-phase analysis (sensitivity analysis).

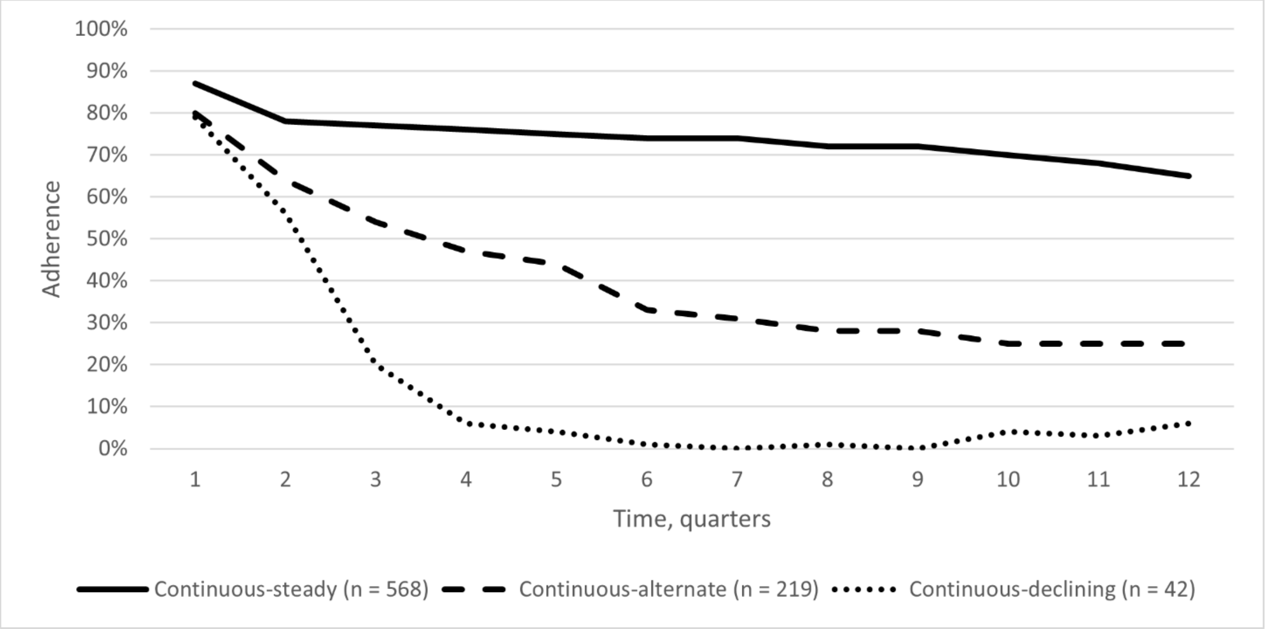

Table S3. Distribution of baseline characteristics of 829 new users of biologic DMARDs in the second-phase analysis (sensitivity analysis).

**Baseline Characteristics**	***Sub-trajectories***			
	**Continuous-steady**	**Continuous-alternate**	**Continuous-declining**	***p*-value**
Overall sample. n (%)	568	219	42	
Gender. n (%)				
Female	418 (73.6)	165 (75.3)	37 (88.1)	0.110
Age. years				
mean (SD)	52.2 (18.4)	51.2 (20.1)	58.0 (17.5)	0.102
Index date year. n (%)				0.380
2010	74 (13.0)	30 (13.7)	5 (11.9)	
2011	100 (17.6)	33 (15.1)	5 (11.9)	
2012	88 (15.5)	46 (21.0)	8 (19.0)	
2013	80 (14.1)	35 (16.0)	11 (26.2)	
2014	118 (20.8)	43 (19.6)	6 (14.3)	
2015	108 (19.0)	32 (14.6)	7 (16.7)	
Comorbidities. n (%)				
Lung disease	10 (1.8)	4 (1.8)	2 (4.8)	0.391
Myocardial infarction	2 (0.4)	0 (0.0)	0 (0.0)	0.631
Other CV diseases	16 (2.8)	6 (2.7)	6 (14.3)	<0.001
Stroke	5 (0.9)	1 (0.5)	0 (0.0)	0.699
Hypertension	14 (2.5)	8 (3.7)	3 (7.1)	0.188
Diabetes	15 (2.6)	8 (3.7)	1 (2.4)	0.734
Fractures	7 (1.2)	3 (1.4)	0 (0.0)	0.754
Depression	0 (0.0)	1 (0.5)	0 (0.0)	0.248
Gastrointestinal ulcer	0 (0.0)	0 (0.0)	0 (0.0)	NA
Other gastrointestinal disorders	6 (1.1)	0 (0.0)	0 (0.0)	0.249
Sjögren's syndrome	3 (0.5)	2 (0.9)	0 (0.0)	0.719
Rheumatoid nodules	0 (0.0)	0 (0.0)	0 (0.0)	NA
Myopathies	1 (0.2)	0 (0.0)	0 (0.0)	0.795
Polyneuropathy	2 (0.4)	0 (0.0)	0 (0.0)	0.631
Additional immune-mediated disorders	36 (6.3)	15 (6.8)	3 (7.1)	0.953
Cancer	9 (1.6)	1 (0.5)	1 (2.4)	0.385
Concomitant therapies. n (%)				
Glucocorticoid	450 (79.2)	175 (79.9)	32 (76.2)	0.862
Non-steroidal anti-inflammatory drugs	369 (65.0)	153 (69.9)	24 (57.1)	0.204
Opioid analgesic	169 (29.8)	61 (27.9)	16 (38.1)	0.411
Conventional synthetic DMARDs	504 (88.7)	186 (84.9)	38 (90.5)	0.297
Index drug. n (%)				
Abatacept	62 (10.9)	19 (8.7)	2 (4.8)	0.327
Etanercept	226 (39.8)	103 (47.0)	19 (45.2)	0.165
Infliximab	10 (1.8)	7 (3.2)	2 (4.8)	0.264
Adalimumab	142 (25.0)	57 (26.0)	7 (16.7)	0.433
Certolizumab pegol	46 (8.1)	13 (5.9)	3 (7.1)	0.584
Golimumab	38 (6.7)	12 (5.5)	6 (14.3)	0.114
Tocilizumab	44 (7.7)	8 (3.7)	3 (7.1)	0.117

CV: cardiovascular; DMARD: disease modifying antirheumatic drugs; n: number; SD: standard deviation
